# Mouse mitochondrial lipid composition is defined by age in brain and muscle

**DOI:** 10.18632/aging.101204

**Published:** 2017-03-21

**Authors:** Amelia K. Pollard, Catharine A. Ortori, Reinhard Stöger, David A. Barrett, Lisa Chakrabarti

**Affiliations:** ^1^ School of Veterinary Medicine and Science, University of Nottingham, Sutton Bonington, LE12 5RD, UK; ^2^ Centre for Analytical Bioscience, School of Pharmacy, University of Nottingham, NG7 2RD, UK; ^3^ Division of Animal Science, School of Biosciences, University of Nottingham, LE12 5RD, UK

**Keywords:** mitochondria, global lipidomics, ageing, mouse, skeletal muscle, brain

## Abstract

Functionality of the lipid rich mitochondrial organelle declines with increased age. Recent advances in lipidomic technologies allowed us to perform a global characterisation of lipid composition in two different tissue types and age ranges. Ultra-high performance liquid chromatography coupled with high resolution mass spectrometry was used to establish and compare mitochondrial lipidomes of brain and skeletal muscle from young (4-11 weeks old) and middle age (78 weeks old) healthy mice. In middle age the brain mitochondria had reduced levels of fatty acids, particularly polyunsaturated fatty acids, while skeletal muscle mitochondria had a decreased abundance of phosphatidylethanolamine, but a pronounced increase of triglyceride levels. Reduced levels of phosphatidylethanolamines are known to decrease mitochondrial membrane fluidity and are connected with accelerated ageing. In mitochondria from skeletal muscle we propose that increased age causes a metabolic shift in the conversion of diacylglycerol so that triglycerides predominate compared with phosphatidylethanolamines. This is the first time mitochondrial lipid content in normal healthy mammalian ageing brain and muscle has been catalogued in such detail across all lipid classes. We identify distinct mitochondrial lipid signatures that change with age, revealing tissue-specific lipid pathways as possible targets to ameliorate ageing-related mitochondrial decline.

## INTRODUCTION

Ageing manifests itself in the progressive decline of biological functions, reducing the capability of an organism to respond to internal and external stress [1]. Features of mitochondrial dysfunction such as reduced respiratory chain efficiency, morphological alterations and mtDNA mutations are entwined with the process of ageing and age-related disease [2, 3]. Rapid advances in lipidomic techniques have led to recent progress in our understanding of the importance of lipids in maintaining lifelong health and changes in disease [4,5]. Mass spectrometry based lipidomics has revealed the complex lipid composition ranging from organelles and cells to tissues and whole organisms [6,7]. Whilst the lipid composition of the mitochondrion has been investigated [8-10] little is known about the changes to the whole mitochondrial lipidome in different tissue types with ageing [11].

Mitochondria are highly specialised organelles with a complex structure consisting of two membranes. Lipids are distributed differently between the inner mitochon-drial membrane and outer mitochondrial membrane. The outer mitochondrial membrane is fluid and contains higher levels of cholesterol and phosphatedylinositol. In contrast, the inner mitochondrial membrane is highly folded, enriched in phosphatidylethanolamines and contains cardiolipin, a component synthesised and restricted to this membrane layer [12]. Mitochondria are characterised by a high, phospphatidylethanolamine and phosphatidylcholine content and low levels of sterols and sphingolipids [13].

Harman first proposed the mitochondrial free radical theory of ageing 60 years ago [14]. This theory postulates that the accumulation of reactive oxygen species (ROS) over the lifespan results in oxidative damage to mitochondrial DNA, proteins and lipids. Lipids are particularly vulnerable to oxidation from ROS since the respiratory chain, a producer of ROS, is embedded within the inner mitochondrial membrane [15]. ROS and free radicals are more soluble in the lipid bilayer and interact with polyunsaturated fatty acids to form highly reactive lipid radicals such as electrophilic aldehydes [16].

Oxidative damage to the mitochondrial lipidome with ageing can cause mitochondrial dysfunction [17,1]. Cardiolipin interacts with proteins of the inner membrane and is vital for processes such as oxidative phosphorylation [18–20], mitochondrial dynamics [21] and apoptosis [22,23]. Reduced levels of cardiolipin not only cause free radical production and lipid peroxidation but also result in decreased mitochondrial functionality [24]. Mitochondrial morphodynamics are regulated by lipid composition with species such as diglycerides, phosphatidylethanolamines, phsophatidyl-serines and cholesterol all being essential to create a negative membrane curvature, required for mitochon-drial fusion [25]. Reductions in the abundance of these lipids within the mitochondria are suggested to cause the enlarged and fragmented mitochondria that are associated with ageing [26].

We set out to evaluate the mitochondrial lipidome through the ageing process in two tissue types that are known to be undergo functional decline during normal ageing, the brain and skeletal muscle, respectively. Certain lipids are synthesised by the organelle itself giving us direct insight into the mechanics of mitochondrial ageing [13]. Lipid content of skeletal muscle is postulated to have a role in longevity but it is only recent advances in the analyses of these types of molecules that allow comprehensive characterisation in mitochondrial fractions [27]. We interrogated the brain and skeletal muscle mitochondria in young (4-11 weeks) mice, the equivalent human age of early adolescence, and old (78 weeks) mice reflecting middle age in humans (approximately 56 years) [28]. We report specific lipid signatures of the ageing brain and skeletal muscle mitochondria.

## RESULTS

### The mitochondrial lipidomes differ distinctly between muscle and brain and change with age

**Figure 1:**
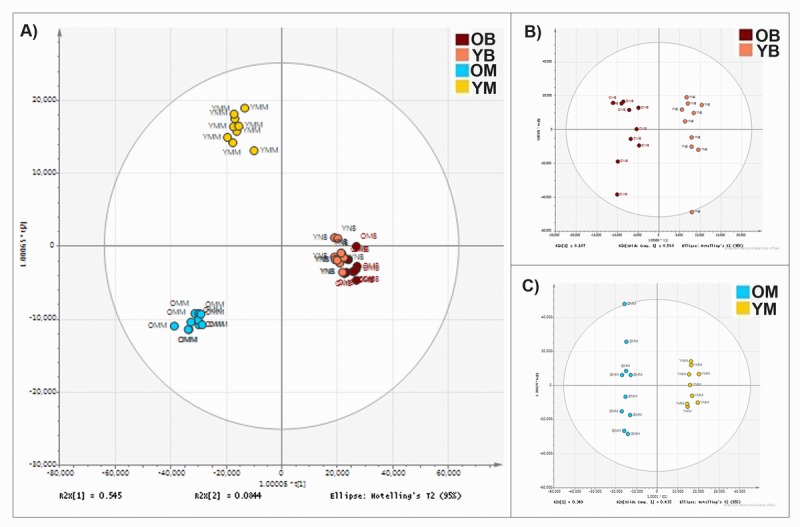
Mitochondrial lipid composition differs between tissue types and changes with age (**A**) Orthogonal partial least square-discriminant analysis (OPLS-DA) of lipids found in the mitochondria of murine brain and skeletal muscle. Separation across the x-axis is according to tissue type. Along the y-axis separation delineates age. There are clear lipid compositional alterations between the mitochondria isolated from the two tissue types and with differences in age of the tissue. (**B**) OPLS-DA of lipid composition of murine brain mitochondria aged 4-11 weeks (pink) (n=10) and 78 weeks (red) (n=10). (**C**) OPLS-DA of the lipid composition of murine muscle mitochondria aged 4-11 weeks (yellow) (n=9) and 78 weeks (blue) (n=10).

We analysed the lipid composition of enriched mitochondrial fractions from young (4-11 weeks) and older (78 weeks) mouse brain and skeletal muscle tissues using UHPLC-HRMS. In total we determined that 8,814 lipids alter in abundance in the mitochondria. Analyses of our datasets show that there are clear tissue specific differences in mitochondrial lipid profiles. Brain and muscle mitochondria each have characteristic compositions, and the lipid composition of each is strongly affected by increased age (Fig. 1). There is little overlap between the most changed lipids in the muscle and brain mitochondria, demonstrating that each tissue ages along independent and characteristic pathways.

### The top lipid changes to the brain and skeletal muscle mitochondria with ageing

**Figure 2:**
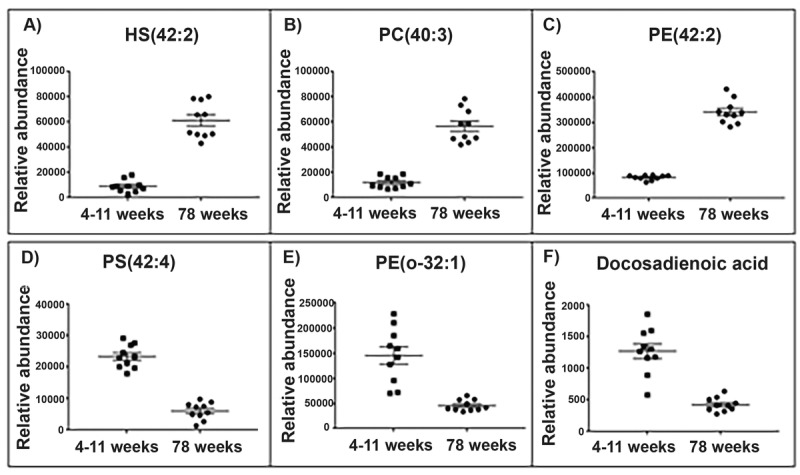
Top lipid species changed with age in the brain mitochondrial lipidome Scatter plots (**A**-**C**) display the top three lipids that increase in abundance in the aged (78 weeks) brain mitochondria compared with young (4-11 weeks) brain mitochondria (old n=10, young n=10). Scatter plots (D-F) display the top three lipids that decrease in abundance in the aged brain mitochondria. (**A**) The lipid 906.632 tentatively identified as hydroxyl sulfatide (42:2) significantly increases in abundance by a fold change of 6.74 in the aged brain mitochondria. (**B**) The lipid *m/z* 826.672 tentatively identified as phosphatidylcholine (40:3) significantly increases in abundance by a fold change of 4.68 in the aged brain mitochondria. (**C**) The lipid *m/z* 810.643 tentatively identified as phosphatidylethanolamine (42:2) significantly increases in abundance by a fold change of 4.17 in the aged brain mitochondria. (**D**) The lipid *m/z* 866.594 tentatively identified as a phosphatidylserine (42:4) significantly decreases in abundance by a fold change of 3.83 in the aged brain mitochondria. (**E**) The lipid *m/z* 732.588 tentatively identified as phosphatidylethanolamine (o-32:1) significantly decreases in abundance by a fold change of 3.19 in the aged brain mitochondria. (**F**) The lipid *m/z* 335.295 tentatively identified as a docosadienoic acid (C22:4) significantly decreases in abundance by a fold change of 3 in the aged brain mitochondria. Scatter plots display abundance ± SEM. We used Wilcoxon rank test and Bonferroni correction (*p*<0.05). Lipids were identified using Lipid Maps and Human Metabolome databases. Refer to Supplementary Tables 3 and 4 for the top 50 lipid changes. Abbreviations: hydroxyl sulfatide (HS), phosphatidylcholine (PC), phosphatidylserine (PS) and phosphatidylethanolamine (PE).

**Figure 3:**
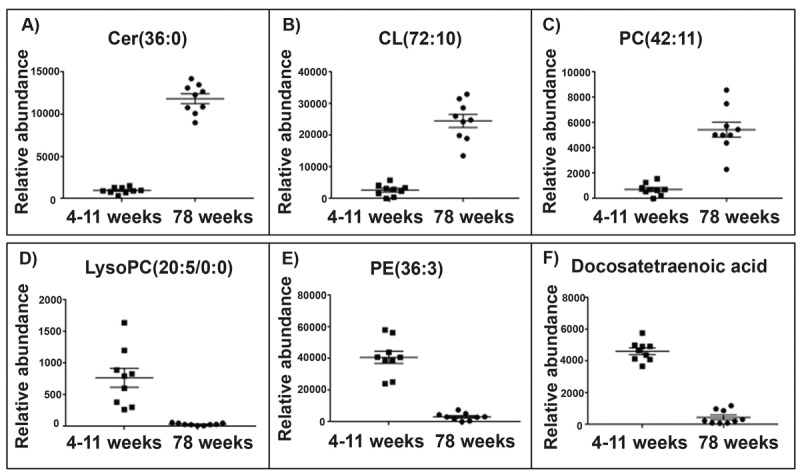
Top lipid species changed with age in the skeletal muscle mitochondrial lipidome Scatter plots (**A**-**C**) display the top three lipids that increase in abundance in aged 78 weeks skeletal muscle mitochondria (n=10) compared with young 4-11 weeks skeletal muscle mitochondria (n=10). The scatter plots (**D**-**F**) display the top three lipids that decrease in abundance in the aged skeletal muscle mitochondria compared to the young skeletal muscle mitochondria. (**A**) The lipid *m/z* 642.568 tentatively identified as ceramide (36:0) increases in abundance by a fold change of 12.37 in the aged skeletal muscle mitochondria. (**B**) The lipid *m/z* 1466.917 tentatively identified as cardiolipin (72:10) increases in abundance by a fold change of 9.31 in the old skeletal muscle mitochondria. (**C**) The lipid *m/z* 874.537 tentatively identified as phosphatidylcholine (42:11) increases in abundance by a fold change of 7.52 in aged skeletal muscle mitochondria. (**D**) The lipid *m/z* 564.305 tentatively identified as lyso-phosphatidylcholine (20:5/0:0) decreases in abundance by a fold change of 23.19 in aged skeletal muscle mitochondria. (**E**) The lipid *m/z* 1504.035 tentatively identified as phosphatidylethanolamine (36:3) decreases in abundance by a fold change of 12.96 in aged skeletal muscle mitochondria. (**F**) The lipid *m/z* 331.263 tentatively identified as docosatetraenoic acid decreases in abundance by a fold change of 10.12 in aged skeletal muscle mitochondria. Scatter plots display the abundance ± SEM. We used the Wilcoxon rank test and Bonferroni correction (*p*<0.15). Lipids were identified using Lipid Maps and Human Metabolome databases. Refer to Supplementary Tables 1 and 2 for the top 50 lipid changes. Abbreviations: ceramide (cer), cardiolipin (CL), phosphatidylcholine (PC), lysophosphatidylcholine (lysoPC) and phosphatidylethanolamine (PE).

With such profound differences between the lipid sets of each tissue type we analysed them separately to find molecular species with the greatest change in abundance per tissue. The top lipids with the highest significant difference (Wilcoxon rank test and Bonferroni correction) and the greatest fold change were selected from each of the brain and skeletal muscle mitochondria LC-MS datasets). Visual representations of the top 6 lipid changes in brain and skeletal muscle mitochondria are shown (Figs. 2 and 3).

Three of the top five lipids to significantly increase in abundance in old brain mitochondria were identified as phosphatidylethanolamines and showed a fold change increase ranging from 3.22 to 4.17. Of the top five lipids to decrease with ageing brain, two lipids were identified as fatty acids. One of these two lipids, with a mass value (*m/z* 335.295), tentatively identified as docosadienoic acid C22:4, has a 3-fold significant decrease in abundance in aged brain mitochondria. The second lipid (*m/z* 307.264), tentatively identified as eicosadienoic acid C20:2, significantly decreases over two-fold in mitochondria of aged brain (Supplementary Table 1).

Of the top five lipids that increase in abundance with ageing in the skeletal muscle mitochondria two lipids were identified as cardiolipins. The lipid *m/z* 1466.917, tentatively identified as cardiolipin (72:10) increases in the aged skeletal muscle mitochondria by a fold change of 12.37. The lipid *m/z* 1693.97, tentatively identified as cardiolipin (70:6), increases over 9-fold (9.31) in abundance in the aged skeletal muscle mitochondria. Of the top five lipids to decrease in abundance in the aged skeletal muscle mitochondria, three lipids were identified as phosphatidylcholine. One of these lipid changes is the polyunsaturated fatty acid *m/z* 331.263, tentatively identified as docosatetraenoic acid, which decreases in abundance in the aged skeletal muscle by a fold change of 10.12 (Supplementary Table 2).

### Fatty acid composition decreases in brain mitochondria from aged animals

**Figure 4:**
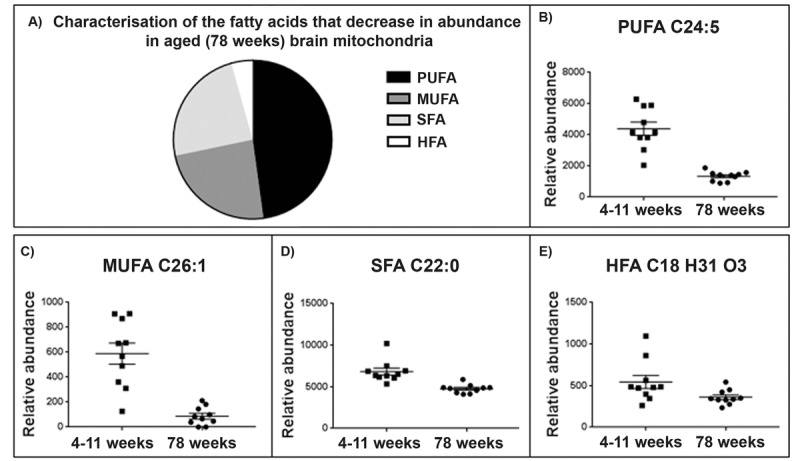
Brain mitochondrial fatty acid composition decreases with ageing (**A)** Characterisation of fatty acid species that decrease in the ageing brain mitochondria. 94% of the fatty acids identified (Supplementary Table 5) decrease with ageing in the brain mitochondria. 48% of the fatty acids found to decrease were identified as polyunsaturated fatty acids (PUFA), 24% were saturated fatty acids (SFA), 24% monounsaturated fatty acids (MUFA) and a small percentage, 4%, were identified as hydroxy fatty acids (HFA). (**C**) Representative scatter plot for the lipid *m/z* 357.2796, tentatively identified as PUFA C22:5. This lipid shows a significant decrease in abundance in 78 week old brain mitochondria (n=10) compared to 4-11 week old brain mitochondria (n=10), *p*=0.048. (**D**) Representative scatter ploy for the lipid *m/z* 393.3732, tentatively identified as a C26.1 MUFA shows a decrease in abundance aged brain mitochondria. (**E**) Representative scatter plot for the saturated fatty acids. The lipid *m/z* 339.3264, tentatively identified as SFA C22:0 decreases in abundance in aged brain mitochondria. (**F**) Representative scatter plot for the lipid *m/z* 295.2274 tentatively identified as HFA C18 H31 O3. This lipid decreases in abundance in aged brain mitochondria. Scatter plots display abundance ± SEM. We used Wilcoxon Rank test and Bonferroni correction. Refer to Supplementary Table 5.

46 fatty acids are identified within our dataset using the Human Metabolome and Lipid Maps databases. This group can be further defined as consisting of poly-unsaturated fatty acids (PUFAs), Monounsaturated fatty acids (MUFAs), saturated fatty acids (SFA) and hydroxyl fatty acids (HFA) (refer to Supplementary Table 3). We see a decrease in the abundance of most of these fatty acids with age in brain mitochondria (Fig. 3): 94% of them decrease in the old brain mitochondria with fold changes in the abundance of the lipids ranging from 0.80 to 27.62 (Fig. 4). Omega-3 PUFAs (ω3PUFAs) decrease and linolenic, docosapentaenoic acid, eicosatrienoic acid and docosahexaenoic acid specifically decrease in abundance in the 78 week old brain mitochondria. MUFAs, SFAs and HFAs also decrease in abundance in the aged brain mitochondria and make up the remaining 52% of the fatty acids identified (fig 4B). Representative scatter plots for each fatty acid classes; PUFA, MUFA, SFA and HFA are shown (Fig. 4C-F).

### Triglycerides increase in aged skeletal muscle mitochondria

**Figure 5:**
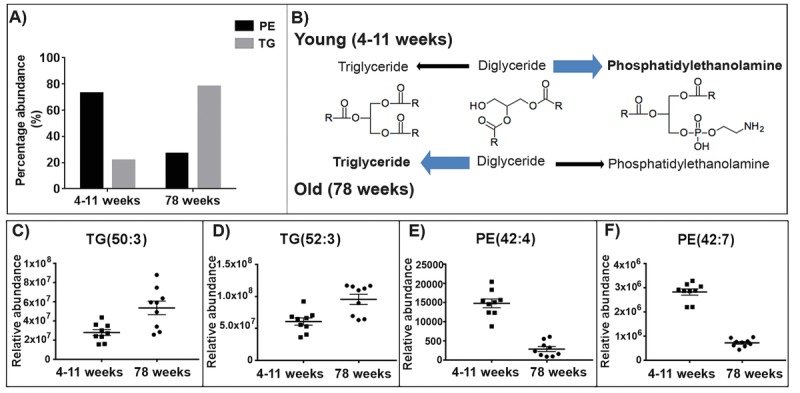
The abundance of triglycerides (TGs) increases in the aged skeletal muscle mitochondria whilst the abundance of phosphatidylethanolamines (PEs) decreases (**A**) 78% of TGs identified increase in abundance with ageing in the skeletal muscle mitochondria. Whilst in the skeletal muscle mitochondria 73% of PEs identified decrease in abundance with ageing. (**B**) Proposed pathways for the lipid changes in the young (4-11 weeks) and old (78 weeks) mitochondria. In the young mitochondria the conversion of diacylglycerols favours the production of phosphatidylethanolamine. Whilst in the old mitochondria the production of diacylglycerols to triglycerides is enhanced. Chemical structures were obtained from Kegg [45]. (**C** and **D**) Representative scatter graphs of three triglycerides that increase in abundance in the aged skeletal muscle mitochondria. (**E** and **F**) Representative scatter graphs of the three PEs that significantly decrease in abundance in the aged skeletal muscle mitochondria (p<0.05). Scatter plots display abundance ± SEM. We used Wilcoxon rank test and Bonferroni correction (4-11 weeks n=9 and 78 weeks n=9). Refer to Supplementary Table 4.

An increase in the abundance of triglycerides (TG) appears to be a characteristic feature of the aged skeletal muscle mitochondrial lipidome (Fig. 5A). Representative scatter plots are shown for two of the triglycerides identified with the greatest fold change (Fig. 5C and 5D). The lipid *m/z* 846.753, tentatively identified as TG (50:3) increases in abundance in the old skeletal muscle mitochondria by a fold change of 1.92. The lipid *m/z* 874.785, tentatively identified as TG (52:3) increases in abundance in the old skeletal muscle mitochondria by a fold change of 1.57 (Supplementary Table 4).

### Phosphatidylethanolamines decrease in aged skeletal muscle mitochondria

There is a reduction in the abundance of phosphatidy-lethanolamines (PEs) in aged (78 weeks) skeletal muscle mitochondria compared to young (4-11 weeks) skeletal muscle mitochondria. 73% of the PE species we identified decrease in abundance in the aged skeletal muscle mitochondria (Fig. 5A). Representative scatter plots are shown for two of the PEs identified with the greatest fold change with ageing (Fig. 5F and 5H). The PEs *m/z* 717.566 and 822.600 both significantly decrease in abundance in the old skeletal muscle mitochondria by fold changes of 7.16 and 5.17, respectively (p<0.05) (Supplementary Table 4).

## DISCUSSION

We demonstrate that the composition of the murine mitochondrial lipidome is different between two energetically active tissues, muscle and brain. We also discovered age-specific changes of mitochondrial lipids in both of these tissues. These mitochondrial lipid signatures describe changes that can classify the tissue and age of the animals.

The phospholipid class has been previously shown to alter with ageing in brain and liver mitochondria [11]. In comparison, our study uses a global lipidomic approach to investigate lipid species from every class of the mitochondrial lipidome during ageing. We uncover in detail the vast extent of tissue-specific mitochondrial lipid changes with age in mouse.

The mouse brain mitochondrial lipidome undergoes a reduction in the abundance of fatty acids with age. With ageing there is an increase in ω6PUFAs and a decrease in ω3PUFAs in the mitochondrial membrane [29]. Changes in the abundance of PUFAs with age alters the fluidity and permeability of mitochondrial membranes [30]. The reduction in ω3PUFAs in the mitochondrial membrane could serve as a protective mechanism to reduce the susceptibility of the mitochondrial membrane to oxidative damage. The normal ageing process is accompanied by a decline in ω3PUFAs in whole brain tissue and increasing dietary intake of ω3PUFAs has been suggested to slow the age-related decline in cognition [31]. Indeed, supplementation of ω3PUFAs influence mitochondrial function by restoring age related reductions in respiration and also improved ATP levels in the brains of aged mice [32].

We find that TG content is increased in the older skeletal muscle mitochondria. TGs are crucial for normal cellular functioning such as energy storage and maintenance of membrane composition, however increased general accumulation of TGs is found in ageing, obesity and type 2 diabetes [33,34]. TG content has previously been shown to increase in mitochondria-rich tissues; heart, liver and skeletal muscle with ageing in rats, supporting the findings from our study [35]. Accumulation of cytosolic levels of TG have been associated with mitochondrial dysfunction due to impaired signalling for fat metabolism [36]. Furthermore, high triglyceride levels have been associated with reduced ATP production and increased ROS production when mitochondrial fusion is impaired [35]. We provide evidence that mitochondrial TG content increases with ageing.

A reduction in the abundance of PE is seen in our ageing skeletal muscle mitochondria lipid set. PE is the second most abundant phospholipid class in the mitochondria and reductions in PEs have a significant impact on mitochondrial functionality through the ageing process [37]. PEs are important for mitochondrial lipid composition and changes to the abundance of PEs, specifically a decline in PEs, within the mitochondrial membrane lead to abnormal mitochondrial morphology, reduced respiratory capacity and impaired fusion and fission [18,38,39]. The abundance of PE is significantly reduced in the *substantia nigra pars compacta* of Parkinson's disease patients compared to controls [40]. Mitochondria from yeast lacking Psd1, the key enzyme that synthesizes PE, show impaired Mgm1-driven mitochondrial fusion along with improper mixing of the lipids in the membrane during fusion [39]. Enlarged mitochondria are a common feature of ageing and may arise as a result of impaired fusion events from reduced PE levels. Indeed, manipulating the abundance of the Psd1 protein has been associated with changes to ageing. Lifespan has been altered in yeast, *Drosophila* and mammalian cells (U2OS and H4) by changing the abundance of PE [41]. PE clearly plays an extremely important role in maintaining healthy mitochondria.

We observe an increase in the abundance of TGs and a decrease in the abundance of PEs with ageing in skeletal muscle mitochondria. TGs and glycerophospho-lipids share two common precursors, fatty acyls and glycerol-3-phosphate. The acylation of glycerol-3-phosphate and fatty acyl-CoA yields diacylglycerol-3-phosphate, more commonly known as phosphatide. The biosynthesis of phosphatide can occur in the endoplasmic reticulum and the outer mitochondrial membrane. Phosphatidate can either be converted to TGs or to glycerophospholipids (such as phosphatidy-lethanolamine). Phosphatidate is dephosphorylated by the enzyme phosphatidate phosphatase yielding diacylgycerols. Diacyglycerol synthesis is an inter-mediate step towards the synthesis of TGs catalysed by the enzyme diglyceride acyltransferase. Membrane phospholipids, phosphatidylcholine and phospha-tidylethanolamine are also synthesised from diacyl-glycerols [42]. We propose that the production of TGs and PEs from diacylglycerols differs with ageing in the skeletal muscle mitochondria. In young mitochondria we see that diacylglycerols may be directed towards glycerophospholipid synthesis. Whilst in the older mitochondria we observe the opposite indicating that TG production from diacylglycerols is enhanced (Fig. 5B).

### Conclusions

Our data show that enriched mitochondrial fractions have characteristic lipidomic profiles specific to the age and type of tissue, echoing our findings in the mitochondrial proteome [43]. We find the brain mitochondrial lipidome is highly modulated with ageing, with fatty acids decreased in older animals. We show that skeletal muscle mitochondria have increased TG content and decreased PE content with age. During the normal lifespan the conversion of diacylglycerols to TGs is favoured over the production of PEs. Tissue-specificity is a factor to be considered carefully in order to be able to properly understand mitochondrial biology within normal ageing.

## MATERIALS AND METHODS

### Mitochondrial preparations

Brain and skeletal muscle tissue were collected from young (4-11 week old) and older (78 weeks) C57BL/6J female mice (Charles River). The mitochondrial fraction was isolated from the tissues as previously described [44].

### Mitochondrial lipid extraction

10 biological replicates of each condition (4-11 weeks brain, 78 weeks brain, 4-11 weeks skeletal muscle and 78 weeks skeletal muscle) were taken and enriched mitochondrial fractions prepared, these underwent lipid extraction. 100 μl of chloroform/methanol (2:1) was added to each sample and mixed for 20 min using a multiplace vortex before undergoing centrifugation (1300 rpm, 10 min) at 4°C. The supernatants were removed to HPLC vials. Isopropanol (50 μl) was added to each vial.

### Lipidomics analysis using accurate mass spectrometry

Accurate mass LC-MS was performed on the mitochondrial lipid extracts (10 μL injection volume) using an Accela LC coupled with an Exactive mass spectrometer (ThermoFisher Scientific, Waltham, USA) in positive and negative electrospray ionisation modes (ESI). An ACE EXL Excel 2μm SuperC18 2.1 x 50mm column equipped with an appropriate guard column was maintained at 40°C with a variable flow rate throughout analysis. The LC gradient program used a water 60%/Acetonitrile 40% (A)-to-water (10%)/acetonitrile (100%)/isopropanol (80%) gradient (B) modified with 0.1% ammonium acetate (30%-100 %B over 12 minutes). Ions were monitored within the range of *m/z* 100 to 1500 (ESI voltage: 3500, capillary temperature: 350°C, scan rate: 250 ms, FT resolution: 25,000). A pooled quality control sample comprising 5 μL from each experimental sample was generated and injected throughout the run. LC-MS data were aligned and exported using the propriety software Progenesis QI (Nonlinear Dynamics, UK).

### Data analysis

A Wilcoxen rank sum test was performed on the data using R (https://www.r-project.org/). Lipid analytes with one or more replicates two standard deviations above or below the mean were removed from the dataset before the Bonferroni correction was carried out. Multivariate analysis- orthogonal partial least square-discriminant analysis (OPLS-DA) was carried out (SIMCA-P 13.0.2 version, Umetrics AB, Umea, Sweden). Lipids were identified using accurate mass determinations by reference to the Lipid Maps and Human Metabolome databases.

## SUPPLEMENTARY MATERIALS TABLE


