# Supplementary material for: Mouse mitochondrial lipid composition is defined by age in brain and muscle
Source: Aging (Albany NY). 2017 Mar 21;9(3):986–95. doi: 10.18632/aging.101204 (PMC5391243; doi:10.18632/aging.101204)
Supplement: Supplementary file 1 [file aging-09-986-s001.pdf]

SUPPLEMENTARY MATERIAL

Supplementary Table 1. The top ten lipids that change in abundance in aged brain mitochondria.

m/z	Retention time (mins)	Adduct	ESI mode	Lipid identity	Wilcoxon rank *p* value	Wilcoxon rank and Bonferroni correction *p* value	Change in abundance with age	Fold change
906.632	5.03	M+H	+	Hydroxylated Sulfatide (42:2)	0.000011	0.024	↑	6.74
826.672	5.02	M+H	+	PC(40:3)	0.000011	0.024	↑	4.68
810.643	5.52	M-H	-	PE(42:2)	0.000011	0.024	↑	4.17
1064.721	6.12	M-H	-	PE(O-44:2)	0.000011	0.024	↑	3.86
856.676	5.8	M+H	+	PE(44:2)	0.000011	0.024	↑	3.22
866.594	5.7	M+H	-	PS(42:4)	0.000011	0.024	↓	-3.83
732.588	5.89	M+H	+	PE (o-32:1)	0.000011	0.024	↓	-3.19
335.295	2.12	M-H	-	Docosadienoic acid C22:4	0.000022	0.048	↓	-3
690.508	3.7	M-H	-	PE(32:0)	0.000011	0.024	↓	-2.25
307.264	1.71	M-H	-	Eicosadienoic acid C20:2	0.000011	0.024	↓	-2.21

The mitochondrial lipidome was compared between young 4-11 weeks (n=10) and old 78 weeks (n=10) brain tissue. The top five lipids with the greatest significant difference and highest fold change increase with ageing were selected. The top five lipids with the greatest significant difference and highest fold change decrease with ageing were selected. Lipids were identified using the Human metabolome database and Lipid maps. Only lipid identities with a mass error of less than 5ppm were selected. Abbreviations: phosphatidylcholine (PC), phosphatidylserine (PS) and phosphatidylethanolamine (PE).

Supplementary Table 2. The top ten lipids that change in abundance in aged skeletal muscle mitochondria.

m/z	Retention time (mins)	Adduct	ESI mode	Lipid identity	Wilcoxon rank *p* value	Wilcoxon rank and Bonferroni correction *p* value	Change in abundance with age	Fold change
642.568	5.25	M-H	-	Cer(36:0)	0.000041	0.128	↑	12.37
1466.917	6.56	M+Na-2H	-	CL(72:10)	0.000041	0.128	↑	9.31
874.537	5.11	M+H	+	PC(42:11)	0.000041	0.128	↑	7.52
1693.97	7.21	Na-Ac+Na	-	CL (70:6)	0.000041	0.128	↑	7.46
876.575	3.45	M+Ac-H	-	PE(42:7)	0.000041	0.128	↑	7.16
564.305	0.81	M+Na	+	LysoPC(20:5/0:0)	0.000041	0.128	↓	-23.19
1504.035	4.16	2M+Na-2H	-	PE(36:3)	0.000041	0.128	↓	-12.96
331.263	1.22	M-H	-	Docosatetraenoic acid	0.000041	0.128	↓	-10.12
862.627	5.39	M+Na	+	PC(40:3)	0.000041	0.128	↓	-7.75
924.671	5.52	M+Ac-H	-	PC(42:4)	0.000041	0.128	↓	-7.32

The mitochondrial lipidome was compared between young 4-11 weeks (n=9) and old 78 weeks (n=9) skeletal muscle tissue. The top five lipids with the greatest significant difference and highest fold change increase with ageing were selected. The top five lipids with the greatest significant difference and highest fold change decrease with ageing were selected. Lipids were identified using the Human metabolome database and Lipid maps. Only lipid identities with a mass error of less than 5ppm were selected. Abbreviations: ceramide (cer), cardiolipin (CL), phosphatidylcholine (PC), lysophosphatidylcholine (lysoPC) and phosphatidylethanolamine (PE).

Supplementary Table 3. The top ten lipids that change in abundance in aged skeletal muscle mitochondria.

m/z	Retention time (mins)	ESI mode	Lipid tentative identification	Fatty acid group	Change in abundance with ageing	Fold change
391.358	3.34	-	C26:2	PUFA	↓	-27.62
361.311	2.27	-	C24:3	PUFA	↓	-9.36
313.078	3.28	-	C20:0	SFA	↓	-6.92
393.373	4.01	-	C26:1	MUFA	↓	-6.75
363.327	2.65	-	C24:2	PUFA	↓	-5.86
293.248	1.51	-	C19:2	PUFA	↓	-5.85
385.311	2.05	-	C26:5	PUFA	↓	-5.53
357.28	1.69	-	C24:5	PUFA	↓	-3.27
387.327	2.35	-	C26:4	PUFA	↓	-3.22
335.295	2.12	-	C22:2	PUFA	↓	-3.00
277.217	1.17	-	C18:3 Linolenic	PUFA	↓	-2.65
359.295	1.92	-	C24:4	PUFA	↓	-2.46
395.389	4.93	-	C26:0	SFA	↓	-2.19
329.248	1.37	-	C22:5 Docosapentaenoic acid	PUFA	↓	-1.99
367.358	4.03	-	C24:0	SFA	↓	-1.93
227.201	1.21	-	C14:0	SFA	↓	-1.75
333.28	1.85	-	C22:3	PUFA	↓	-1.75
279.233	1.36	-	Linoelic	PUFA	↓	-1.71
305.248	1.47	-	C20:3 Eicosatrienoic acid	PUFA	↓	-1.67
331.264	1.57	-	C22:4	PUFA	↓	-1.66
337.311	1.53	-	C22:1	MUFA	↓	-1.62
199.17	0.88	-	C12 SFFA	SFA	↓	-1.55
355.264	1.47	-	C24:6	PUFA	↓	-1.54
303.233	1.32	-	C20:4 Arachadonic acid	PUFA	↓	-1.52
317.248	1.45	-	C21:5	PUFA	↓	-1.52
295.227	0.61	-	C18 H31 O3	HFA	↓	-1.51
269.249	1.73	-	C17:0 Heptadecanoic acid	SFA	↓	-1.49
255.233	1.55	-	C16:0 Palmitic	SFA	↓	-1.46
339.326	3.2	-	C22:0	SFA	↓	-1.43
241.217	1.37	-	C15:0 Pentadecanoic acid	SFA	↓	-1.40
311.295	2.5	-	C20:1	MUFA	↓	-1.40
239.201	1.15	-	C15:1	MUFA	↓	-1.38
327.233	1.21	-	C22:6 Docosahexaenoic acid	PUFA	↓	-1.34

365.342	3.22	-	C24:1	MUFA	↓	-1.34
281.248	1.61	-	C18:1 Oleic	MUFA	↓	-1.28
225.186	0.96	-	C14:1	MUFA	↓	-1.23
253.217	1.28	-	C16:1 Palmitoleic acid	MUFA	↓	-1.19
267.233	1.45	-	C17:1 Heptadecenoic acid	MUFA	↓	-1.16
309.28	2.01	-	C20:2	PUFA	↓	-1.14
297.28	2.22	-	C19:0	SFA	↓	-1.06
293.212	0.71	-	C18 H29 O3	HFA	↓	-0.80
295.264	1.81	-	C19:1	MUFA	↑	1.03
325.311	2.84	-	C21:0	SFA	↑	1.03
323.295	2.27	-	C21:1	MUFA	↑	1.43

48 fatty acids were identified in the list of lipid analytes. The average abundance for each fatty acid in the young (4-11 weeks) and aged (78 weeks) murine brain mitochondria are listed. 43 out of the 48 fatty acids decrease in abundance in the old brain mitochondria compared to the young brain mitochondria. A high proportion of the decreased fatty acids were polyunsaturated (PUFA). Monounsaturated (MUFA), saturated fatty acids (SFA). Two hydroxy-fatty acids (HFA) were in this group.

Supplementary Table 4. Representative lipid demonstrating the observed increase in abundance of triglycerides (TGs) and decrease in abundance of phosphatidylethanolamines (PEs) with ageing in the skeletal muscle mitochondria.

m/z	**Retention time (mins)**	**Adduct**	**ESI mode**	**Lipid identity**	**Fold change**
846.753	8.30	M+NH4	+	TG(50:3)	1.92
874.785	9.02	M+NH4	+	TG(52:3)	1.57
822.600	5.63	M-H	-	PE(42:4)	-5.17
764.523	4.20	M-H	-	PE(38:5)	-3.92

The top three identified lipids with the greatest fold change were selected for the representative scatter plots (Fig. 6).
